# Quantifying pH-induced changes in plasma strong ion difference during experimental acidosis: clinical implications for base excess interpretation

**DOI:** 10.1152/japplphysiol.00917.2023

**Published:** 2024-02-29

**Authors:** Lorenzo Giosa, Francesco Zadek, Mattia Busana, Giovanna De Simone, Serena Brusatori, Martin Krbec, Frantisek Duska, Paolo Brambilla, Alberto Zanella, Alessandra Di Masi, Pietro Caironi, Emanuele Perez, Luciano Gattinoni, Thomas Langer

**Affiliations:** ^1^Department of Critical Care Medicine, Guy’s and St. Thomas’ National Health Service Foundation Trust, London, United Kingdom; ^2^Centre for Human and Applied Physiological Sciences, King’s College London, London, United Kingdom; ^3^Department of Medicine and Surgery, University of Milano-Bicocca, Monza, Italy; ^4^Department of Anesthesiology, University Medical Center Göttingen, Göttingen, Germany; ^5^Department of Sciences, Roma Tre University, Rome, Italy; ^6^Department of pathophysiology and Transplantation, University of Milan, Milan, Italy; ^7^Department of Anesthesia and Intensive Care Medicine, The Third Faculty of Medicine, Charles University and FNKV University Hospital, Prague, Czechia; ^8^Department of Anesthesia and Critical Care, AOU S. Luigi Gonzaga, Turin, Italy; ^9^Department of Oncology, University of Turin, Turin, Italy; ^10^Department of biomedical and neuromotor sciences, Headquarter of Human physiology, University of Bologna, Bologna, Italy; ^11^Department of Anesthesia and Intensive Care Medicine, Niguarda Ca’ Granda, Milan, Italy

**Keywords:** albumin, hemoglobin, noncarbonic whole blood buffer value, plasma strong ion difference, whole blood base excess

## Abstract

It is commonly assumed that changes in plasma strong ion difference (SID) result in equal changes in whole blood base excess (BE). However, at varying pH, albumin ionic-binding and transerythrocyte shifts alter the SID of plasma without affecting that of whole blood (SID_wb_), i.e., the BE. We hypothesize that, during acidosis, *1*) an expected plasma SID (SID_exp_) reflecting electrolytes redistribution can be predicted from albumin and hemoglobin’s charges, and *2*) only deviations in SID from SID_exp_ reflect changes in SID_wb_, and therefore, BE. We equilibrated whole blood of 18 healthy subjects (albumin = 4.8 ± 0.2 g/dL, hemoglobin = 14.2 ± 0.9 g/dL), 18 septic patients with hypoalbuminemia and anemia (albumin = 3.1 ± 0.5 g/dL, hemoglobin = 10.4 ± 0.8 g/dL), and 10 healthy subjects after in vitro-induced isolated anemia (albumin = 5.0 ± 0.2 g/dL, hemoglobin = 7.0 ± 0.9 g/dL) with varying CO_2_ concentrations (2–20%). Plasma SID increased by 12.7 ± 2.1, 9.3 ± 1.7, and 7.8 ± 1.6 mEq/L, respectively (*P* < 0.01) and its agreement (bias[limits of agreement]) with SID_exp_ was strong: 0.5[−1.9; 2.8], 0.9[−0.9; 2.6], and 0.3[−1.4; 2.1] mEq/L, respectively. Separately, we added 7.5 or 15 mEq/L of lactic or hydrochloric acid to whole blood of 10 healthy subjects obtaining BE of −6.6 ± 1.7, −13.4 ± 2.2, −6.8 ± 1.8, and −13.6 ± 2.1 mEq/L, respectively. The agreement between ΔBE and ΔSID was weak (2.6[−1.1; 6.3] mEq/L), worsening with varying CO_2_ (2–20%): 6.3[−2.7; 15.2] mEq/L. Conversely, ΔSID_wb_ (the deviation of SID from SID_exp_) agreed strongly with ΔBE at both constant and varying CO_2_: −0.1[−2.0; 1.7], and −0.5[−2.4; 1.5] mEq/L, respectively. We conclude that BE reflects only changes in plasma SID that are not expected from electrolytes redistribution, the latter being predictable from albumin and hemoglobin’s charges.

**NEW & NOTEWORTHY** This paper challenges the assumed equivalence between changes in plasma strong ion difference (SID) and whole blood base excess (BE) during in vitro acidosis. We highlight that redistribution of strong ions, in the form of albumin ionic-binding and transerythrocyte shifts, alters SID without affecting BE. We demonstrate that these expected SID alterations are predictable from albumin and hemoglobin’s charges, or from the noncarbonic whole blood buffer value, allowing a better interpretation of SID and BE during in vitro acidosis.

## Graphical abstract

**Figure:**
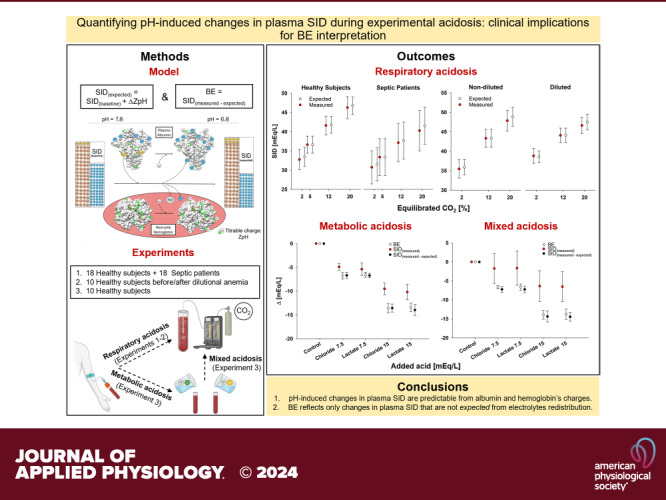

## INTRODUCTION

Whole blood base excess (BE) is the Pco_2_-independent variable that quantitatively describes metabolic acidosis in vitro (1). It is calculated as the deviation of buffer base (BB) from its normal value (2). The buffer base represents the total negative charge on blood buffers, namely bicarbonate, proteins, and phosphate species (3).

Under all circumstances, the negative charge of the buffer base is balanced by an equal and positive strong ion difference (SID), i.e., the difference between strong cations (mainly Na^+^, K^+^, Ca^2+^, and Mg^2+^) and anions (mainly Cl^−^, Lac^−^, and unmeasured anions).

For electroneutrality to be preserved, when SID deviates from its normal value (e.g., for addition of strong ions to blood), the buffer base must change of an equal amount (3).

Accordingly, in solutions with constant concentration of total weak acids, the following statement holds true: since BE = ΔBB and ΔSID = ΔBB, then ΔSID = BE. Briefly, any change in strong ion difference should be reflected by an equal base excess. For instance, in a patient with hyperchloremia where SID decreases by 10 mEq/L, BE should decrease by 10 mEq/L. This assumption is now part of several acid-base models attempting to partition the base excess at the bedside (4–9). However, this approach has been largely criticized, in that it represents an inappropriate application of the Stewart’s plasma acid-base model to the whole blood concept of BE (10, 11). Indeed, according to Stewart, SID is independent of pH, and rather determines pH when deviating from normal (12). However, the SID that is measured in plasma is not pH-independent in whole blood: the plasma concentration of strong ions changes at varying pH, due to binding of electrolytes to albumin (13–15) and, more importantly, to shifts of electrolytes and water between plasma and red cells (16, 17). This physiological redistribution of strong ions does not alter the total pool of blood charges (i.e., the SID of whole blood), and thereby should not affect BE. For instance, when CO_2_ increases in blood, the reducing pH forces water and chloride into the red cells, thereby increasing plasma SID (18, 19). However, BE does not concomitantly change, as electrolytes do not physically leave blood (the whole blood SID remains constant), but simply change compartment (20). Similarly, during metabolic acidosis, addition of acid to blood reduces BE by an equal amount (e.g., addition of 10 mEq/L of lactic acid reduces BE by 10 mEq/L), but the total reduction in plasma SID is lower, as some chloride, lactate, and water are forced into the red cells by the decreasing pH (21–23).

Although these concepts have been largely explored in the past, a quantification of the expected changes in plasma SID at varying pH is lacking, and it is unknown how this may influence the relationship between plasma SID and whole blood BE.

**Figure 1.**
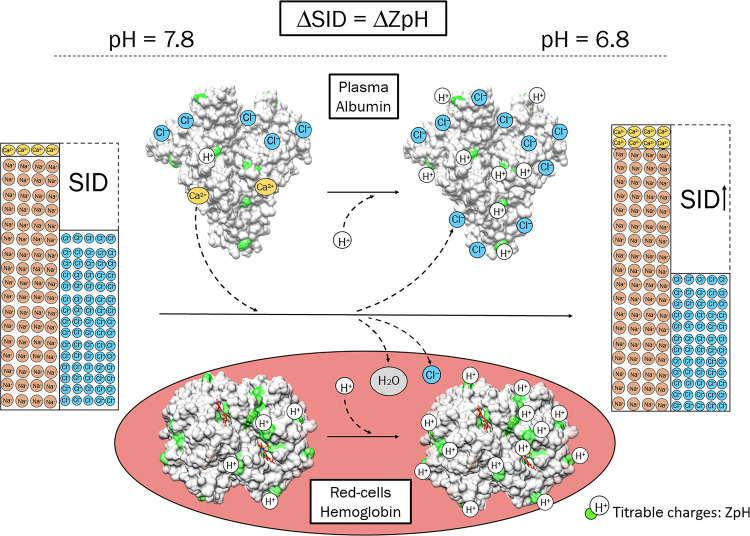
Changes in strong ion difference (SID) at varying pH. This figure depicts the predicted increase in *Z*_pH_ on albumin (heart shaped, *top* part of the figure) and hemoglobin (inside a schematic red cell, *bottom* part of the figure) when pH decreases from 7.8 to 6.8. The hypothesized effect of pH-induced changes in *Z*_pH_ on SID is also highlighted: the increase in albumin *Z*_pH_ is associated with release of bound calcium, and increased binding of chloride. Concomitantly, the increase in hemoglobin *Z*_pH_ drives chloride and water into red cells. Both phenomena determine an increase in SID. Based on previously published experiments (14, 17), we hypothesize that the overall change in SID to be expected for a given change in pH in whole blood equals the predictable change in albumin and hemoglobin’s *Z*_pH_. Implications of such hypothesis are discussed in the text. Note that, for the sake of simplicity, the figure neglects potassium and magnesium, whose pH dependence is quantitatively less important than chloride, calcium, and sodium. Chloride binding to hemoglobin is also not shown since irrelevant to this paper. Albumin and hemoglobin were drawn using UCSF ChimeraX package (27).

The aim of this in vitro study is therefore twofold: first, to simplify the quantification of electrolytes/water redistribution at varying pH, currently prerogative of complex computerized acid-base models (11, 24). Here, we hypothesize that this phenomenon can be modeled as a simple function of the titrable (pH-dependent) charge on albumin and hemoglobin (25), the predominant noncarbonic buffers in blood (26) (Fig. 1). On these grounds, we will introduce the novel concept of expected strong ion difference (SID_exp_), defined as the predictable plasma SID that is expected to result from electrolytes redistribution at varying pH. Second, we aim to compare changes in SID and BE during metabolic and mixed acidosis. We hypothesize that only changes in plasma SID that are not explained by expected electrolytes and water shifts will reflect changes in whole blood SID, and thereby be paralleled by an equal BE.

## METHODS

### Study Populations

We tested our hypotheses in three in vitro experiments investigating: *1*) Respiratory acidosis in isolated plasma and whole blood of 18 healthy subjects and 18 age-matched septic patients with anemia and hypoalbuminemia (18); *2*) Respiratory acidosis in whole blood of 10 healthy subjects before and after in vitro-induced isolated anemia; *3*) Respiratory, metabolic, and mixed acidosis in whole blood of 10 healthy subjects (28).

Detailed methods for *experiments 1* and *3* were previously published elsewhere (18, 28). All experiments were approved by the local ethical committee (Comitato Etico Milano Area 2, Protocol 124_2018bis).

### Procedures and Measurements

#### Experiment 1.

A venous blood sample was assessed for hemoglobin, albumin, phosphate, and magnesium concentrations (Cobas c-702, Roche, Switzerland). Isolated plasma was then separated from whole blood by centrifugation. Subsequently, plasma and whole blood samples were equilibrated (Equilibrator, RNA Medical) with heated (37°C) and oxygenated (21% O_2_) gas mixtures containing four CO_2_ concentrations (2, 5, 12, and 20%). Blood gases and electrolytes were assessed at every equilibrated CO_2_ (ABL 800 FLEX Radiometer, Denmark). Further details are available in the study by Langer et al. (18).

#### Experiment 2.

Venous blood was collected with a vacuum technique in lithium-heparin tubes (Vacuette Plasma Lithium Heparin 4 mL Tubes, Greiner Bio-One, Kremsmünster, AT). Each subject provided six tubes, of which one was used for preprotocol analyses, one was used as “nondiluted” sample, and three underwent centrifugation at 4°C at 3,000 rpm for 12 min to obtain isolated plasma. Plasma was then mixed with whole blood in the remaining tube, obtaining a “diluted” sample with anemia (half the initial hematocrit) and normoalbuminemia. Diluted and nondiluted whole blood samples were assessed for hemoglobin, albumin, phosphate, and magnesium concentrations (Cobas c-702, Roche, Switzerland), then equilibrated (Equilibrator, RNA Medical) with heated (37°C) and oxygenated (21% O_2_) gas mixtures containing three CO_2_ concentrations (2, 12, and 20%). Blood gases and electrolytes were measured at every equilibrated CO_2_ (ABL 800 FLEX Radiometer, Denmark).

#### Experiment 3.

A venous blood sample was assessed for blood count, albumin, phosphate, and magnesium concentrations (Cobas c-702, Roche, Switzerland), then mixed with five stock electrolytes solutions to obtain:

- A control sample (SID similar to normal plasma: “Ctr”);
- Two samples with hyperchloremic acidosis (addition of ∼ 7.5 and 15 mEq/L of hydrochloric acid: ‘Cl 7.5’ and ‘Cl 15’);
- Two samples with lactic acidosis (addition of ∼ 7.5 and 15 mEq/L of lactic acid: ‘Lac 7.5’ and ‘Lac 15’).

After mixing, all samples were equilibrated (Equilibrator, RNA Medical) with heated (37°C) and oxygenated (21% O_2_) gas mixtures containing ∼10 different CO_2_ concentrations (from 2 to 20%), then assessed for blood gases and electrolytes (ABL 90 Radiometer, Denmark). Further details are available in the study by Krbec et al. (28).

#### Baseline.

In every experiment, a baseline step was defined as the one with pH closest to 7.4 and Pco_2_ closest to 40 mmHg. Specifically:

1)  *Experiment 1*: samples equilibrated with 5% CO_2_.
2)  *Experiment 2*: samples equilibrated with 12% CO_2_.
3)  *Experiment 3*: the control samples (Ctr) with PCO_2_ closest to 40 mmHg.

Changes in measured and calculated variables from baseline will be referred to as Δ throughout the manuscript.

### Calculations

#### Variables calculated in all experiments.

SID was calculated from measured plasma electrolytes as:

(*1*) $$\text{SID}=[{\text{Na}}^{+}]+[{\text{K}}^{+}]+[{\text{Ca}}^{2+}]+[{\text{Mg}}^{2+}]-[{\text{Cl}}^{-}]-[{\text{Lac}}^{-}]$$

where brackets indicate concentrations (mEq/L) of sodium (Na^+^), potassium (K^+^), ionized calcium (Ca^2+^), magnesium (Mg^2+^), chloride (Cl^−^), and lactate (Lac^−^). Magnesium concentration was available at baseline only and was considered constant throughout the experiments.

Albumin and hemoglobin’s titrable charges (*Z*_pH_) in mEq/L were computed from Watson (25) as:

(*2*) $$Z_{\text{pH}}=c\cdot \frac{n\cdot 10^{-\text{pH}}}{10^{-6.75}+10^{-\text{pH}}}$$

where *c* is the concentration of the protein in mMol/L, pH refers to plasma for albumin and red cells for hemoglobin (16), *n* is the number of titrable groups on each protein (29, 30), and 6.75 is their average dissociation constant (31) (Supplemental Material; see https://doi.org/10.6084/m9.figshare.24903429.v1, Supplemental Fig. S1; see https://doi.org/10.6084/m9.figshare.24903384, and Supplemental Fig. S2; see https://doi.org/10.6084/m9.figshare.24903414).

The expected SID (SID_exp_) was calculated as:

(*3*) $${\text{SID}}_{\text{exp}}={\text{SID}}_{\left(\text{baseline}\right)}+\Delta Z_{\text{pH }}$$

where *Z*_pH_ refers to albumin in isolated plasma, and to albumin + hemoglobin in whole blood.

#### Additional variables calculated in experiment 3.

Participant’s specific noncarbonic whole blood buffer values (β) were obtained as the opposite of the first derivative of the $\mathrm{HC}O_{3}^{-}$/pH curve during CO_2_ tonometry as previously described (28):

(*4*) $$\beta =-\frac{\text{d}\left[{\text{HCO}}_{3}^{-}\right]}{\text{dpH}}.$$

An alternative expected SID derived from β (SID_exp(β)_) was calculated as:

(*5*) $${\text{SID}}_{\text{exp}(\text{β})}={\text{ SID}}_{\left(\text{baseline}\right)}\;-\text{ β}\cdot (\text{pH}-{\text{pH}}_{\left(\text{baseline}\right)}).$$

Whole blood BE was computed with Lang and Zander’s equation (32) using participants’ specific β values (28):

(*6*) $$\text{BE}=r\cdot \left[\left({\text{HCO}}_{3}^{-}-24.26\right)+\text{β}\cdot \left(\text{pH}-7.4\right)\right]-s$$

where *r* is the distribution ratio of bicarbonate, calculated as 1 – 0.0143·[Hb] ([Hb] being hemoglobin concentration in g/dL), whereas *s* represents the effect of hemoglobin saturation on BE, and is calculated as 0.2·[Hb]·(1 – sO_2_), with sO_2_ being the fraction of saturated hemoglobin.

Changes in whole blood SID (ΔSID_wb_) were calculated as:

(*7*) $$\Delta {\text{SID}}_{\text{wb}}=r\;\cdot (\text{SID}-{\text{SID}}_{\exp\;\left(\text{β}\right)}).$$

#### Simplified model.

A simplified model was built using calculations recommended by the Clinical and Laboratory Standards Institute (CLSI) (33) and available in current blood gas analyzers. Specifically, in all experiments we calculated the buffer value of whole blood as:

(*8*) $$\beta_{\text{CLSI}}=\;1.43\cdot [\text{Hb}]+7.7.$$

We then used β_CLSI_ to calculate an expected SID (SID_exp(CLSI)_) as:

(*9*) $${\text{SID}}_{\text{exp}(\text{CLSI})}={\text{SID}}_{\left(\text{baseline}\right)}-\beta_{\text{CLSI}}\cdot (\text{pH}-{\text{pH}}_{\left(\text{baseline}\right)}).$$

Analogous to *Eqs. 6* and*7*, in *experiment 3* we also calculated whole blood base excess and changes in whole blood SID using CLSI standards:

(*10*) $${\text{BE}}_{\text{CLSI}}=r\cdot \left[\left({\text{HCO}}_{3}^{-}-24.26\right)+\beta_{\text{CLSI}}\cdot \left(\text{pH}-7.4\right)\right]$$

(*11*) $$\Delta {\text{SID}}_{\text{wb}(\text{CLSI})}=\;r\;\cdot (\text{SID}-{\text{SID}}_{\exp\;\left(\text{CLSI}\right)}).$$

### Statistical Analysis

All data are reported as means ± standard deviation (SD). Normality of distributions was assessed with histograms and QQ plots. In all experiments, Bland–Altman analysis was used to describe the agreement (expressed as mean bias [limits of agreement]) between SID and SID_exp_, and SID and SID_exp(CLSI)_ at varying CO_2_. In *experiment 3*, we also assessed the agreement between SID and SID_exp(β)_, ΔBE and ΔSID, ΔBE and ΔSID_wb_, ΔBE_CLSI_ and ΔSID, and ΔBE_CLSI_ and ΔSID_wb(CLSI)_. Agreements were considered clinically acceptable if bias was within ±1 mEq/L and maximal difference <3 mEq/L (34). Between-group differences (e.g., healthy subjects vs. patients with sepsis in *experiment 1*) were assessed with Student’s *t* test. Pearson’s *r* coefficient was used to describe associations between variables. Two-tailed *P* < 0.05 was considered statistically significant. The graphs were formulated with SigmaPlot v.16.0. R-4.2.2 was used for statistical computing.

## RESULTS

### Experiment 1

Albumin and hemoglobin were higher in healthy subjects than in septic patients: 4.8 ± 0.2 versus 3.1 ± 0.5 g/dL and 14.2 ± 0.9 versus 10.4 ± 0.8 g/dL, respectively (both *P* < 0.01).

**Figure 2.**
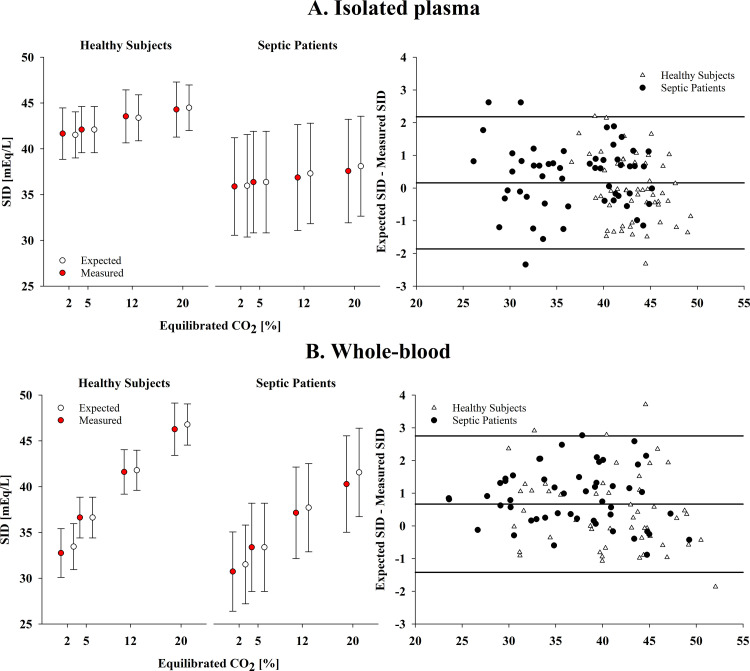
Measured vs. expected strong ion difference (SID) during respiratory acidosis (*experiment 1*). The figure displays mean values of measured and expected SID at every equilibrated CO_2_ (*left*) and their agreement through a Bland–Altman plot (*right*) in isolated plasma (*A*) and whole blood (*B*) of healthy subjects and septic patients. The step with 5% equilibrated CO_2_ (i.e., the baseline step) was not considered in the Bland–Altman analysis, as the measured and expected SID are equal by definition.

In isolated plasma, from 2 to 20% CO_2_, the measured strong ion difference (SID) increased by 2.9 ± 1.0 mEq/L in healthy subjects, and 2.1 ± 0.7 mEq/L in septic patients (*P* < 0.01). The agreement with the predicted strong ion difference (SID_exp_) was strong: 0.0 [−2.0; 2.0] mEq/L and 0.4 [−1.6; 2.4] mEq/L, respectively (Fig. 2*A*).

In whole blood (Table 1), from 2 to 20% CO_2_, the measured plasma strong ion difference (SID) increased by 12.7 ± 2.1 mEq/L in healthy subjects, and 9.3 ± 1.7 mEq/L in patients(*P* < 0.01). The agreement with the predicted strong ion difference (SID_exp_) was strong: 0.5 [−1.9; 2.8] mEq/L and 0.9 [−0.9; 2.6] mEq/L, respectively (Fig. 2*B*).

**Table 1.** Whole blood CO_2_ tonometry in experiment 1

	Healthy Subjects (*n* = 18)				Patients with Sepsis (*n* = 18)			
Variable	2%	5%	12%	20%	2%	5%	12%	20%
PCO_2_	18.2 ± 2.1	32.1 ± 3.4	68.3 ± 4.9	124.4 ± 10.2	18.3 ± 2.0	29.6 ± 2.7	67.2 ± 5.7	122.7 ± 11.0
pH	7.61 ± 0.04	7.46 ± 0.03	7.24 ± 0.03	7.07 ± 0.03	7.56 ± 0.11	7.41 ± 0.10	7.17 ± 0.09	6.98 ± 0.09
HCO_3_^−^	18.0 ± 1.2	22.8 ± 1.8	29.6 ± 1.6	35.7 ± 2.0	16.8 ± 4.3	19.5 ± 5.2	25.1 ± 5.4	29.7 ± 5.6
Na^+^	137.3 ± 2.1	138.9 ± 2.0	140.7 ± 2.1	142.7 ± 2.1	138.7 ± 5.1	138.9 ± 5.1	140.4 ± 5.2	141.7 ± 5.3
K^+^	4.23 ± 0.32	4.25 ± 0.36	4.32 ± 0.28	4.48 ± 0.39	4.35 ± 0.53	4.32 ± 0.57	4.48 ± 0.70	4.57 ± 0.58
Ca^++^	1.09 ± 0.05	1.17 ± 0.05	1.28 ± 0.05	1.36 ± 0.06	1.05 ± 0.07	1.10 ± 0.08	1.17 ± 0.08	1.22 ± 0.09
Mg^++^	2.10 ± 0.14	2.11 ± 0.15	2.11 ± 0.15	2.11 ± 0.15	2.00 ± 0.41	2.05 ± 0.45	2.05 ± 0.45	2.05 ± 0.45
Cl^−^	111 ± 2	109 ± 2	106 ± 2	104 ± 2	112 ± 4	110 ± 4	108 ± 4	107 ± 4
Lac^−^	1.9 ± 0.5	1.7 ± 0.6	1.7 ± 0.5	1.7 ± 0.5	3.8 ± 2.7	3.6 ± 2.8	3.6 ± 2.7	3.5 ± 2.7
SID	32.8 ± 2.7	36.6 ± 2.2	41.6 ± 2.4	46.3 ± 2.9	30.7 ± 4.3	33.4 ± 4.8	37.1 ± 5.0	40.3 ± 5.3
*Z*_pH (Alb)_	1.4 ± 0.1	1.9 ± 0.1	2.8 ± 0.2	3.7 ± 0.2	1.0 ± 0.2	1.4 ± 0.3	2.1 ± 0.4	2.7 ± 0.5
*Z*_pH (Hb)_	11.2 ± 1.1	13.7 ± 1.1	17.9 ± 1.2	22.9 ± 1.5	8.7 ± 1.4	10.7 ± 1.4	14.3 ± 1.5	17.4 ± 1.7
SID_exp_	33.5 ± 2.5	36.6 ± 2.2	41.8 ± 2.2	46.8 ± 2.3	31.5 ± 4.3	33.4 ± 4.8	37.7 ± 4.8	41.6 ± 4.8

Values are means ± SD. SID, strong ion difference; SID_exp_, expected plasma SID; *Z*_pH_, titrable charges.

Supplemental Fig. S3 (see https://doi.org/10.6084/m9.figshare.24899334.v2) reports changes in single electrolytes during acidosis.

### Experiment 2

Albumin was similar in diluted and nondiluted samples (5.0 ± 0.2 vs. 5.0 ± 0.3 g/dL, *P* > 0.99), whereas hemoglobin was significantly lower in the former (7.0 ± 0.9 vs. 14.1 ± 1.7 g/dL, *P* < 0.01).

**Figure 3.**
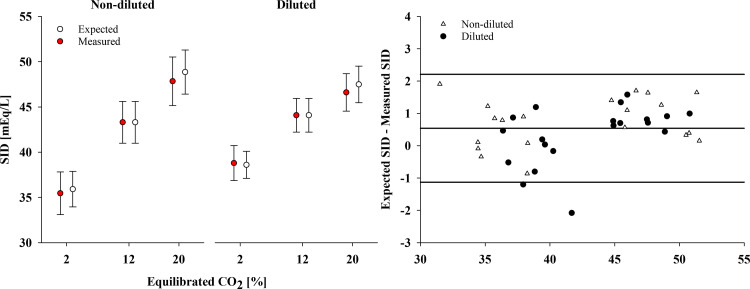
Measured vs. expected strong ion difference (SID) during respiratory acidosis (*experiment 2*). *Left*: mean values of measured and expected SID at every equilibrated CO_2_ in nondiluted and diluted samples. *Right*: their agreement through a Bland–Altman plot. The step with 12% equilibrated CO_2_ (i.e., the baseline steps) was not considered in the Bland–Altman analyses, as the measured and expected SID are equal by definition.

As shown in Table 2, from 2 to 20% CO_2_, the measured strong ion difference (SID) increased by 12.4 ± 2.0 mEq/L in nondiluted samples, and 7.8 ± 1.6 mEq/L in the diluted samples (*P* < 0.01). The agreement with the predicted strong ion difference (SID_exp_) was strong: 0.7 [−0.8; 2.2] mEq/L and 0.3 [−1.4; 2.1] mEq/L, respectively (Fig. 3).

**Table 2.** Whole blood CO_2_ tonometry in experiment 2

	Diluted (*n* = 10)			Undiluted (*n* = 10)		
Variable	2%	12%	20%	2%	12%	20%
PCO_2_	18.6 ± 4.8	66.9 ± 3.7	118.4 ± 9.3	19.9 ± 3.3	64.2 ± 5.3	121.2 ± 8.8
pH	7.69 ± 0.08	7.25 ± 0.03	7.06 ± 0.04	7.60 ± 0.05	7.27 ± 0.03	7.08 ± 0.03
HCO_3_^−^	22.2 ± 2.3	29.6 ± 2.3	33.5 ± 2.3	19.6 ± 2.1	29.8 ± 2.2	36.1 ± 2.7
Na^+^	139.7 ± 1.2	141.6 ± 1.2	142.6 ± 1.3	139.2 ± 1.4	142.0 ± 1.6	143.8 ± 1.6
K^+^	3.97 ± 0.23	3.98 ± 0.28	4.06 ± 0.25	4.10 ± 0.23	4.12 ± 0.27	4.22 ± 0.23
Ca^++^	1.03 ± 0.03	1.28 ± 0.03	1.38 ± 0.02	1.09 ± 0.03	1.28 ± 0.03	1.37 ± 0.03
Mg^++^	2.05 ± 0.12	2.07 ± 0.13	2.09 ± 0.14	2.02 ± 0.10	2.06 ± 0.10	2.10 ± 0.11
Cl^−^	107 ± 2	104 ± 2	103 ± 2	110 ± 2	105 ± 2	103 ± 2
Lac^−^	1.5 ± 0.3	1.4 ± 0.2	1.3 ± 0.2	1.9 ± 0.4	1.7 ± 0.4	1.6 ± 0.4
SID	38.8 ± 1.9	44.1 ± 1.9	46.6 ± 2.1	35.5 ± 2.4	43.3 ± 2.3	47.8 ± 2.7
*Z*_pH (Alb)_	1.2 ± 0.2	2.9 ± 0.1	4.0 ± 0.3	1.5 ± 0.2	2.8 ± 0.2	3.9 ± 0.3
*Z*_pH (Hb)_	4.8 ± 0.7	8.7 ± 1.1	11.0 ± 1.3	11.0 ± 1.3	17.1 ± 1.9	21.5 ± 2.4
SID_exp_	38.6 ± 1.5	44.1 ± 1.9	47.5 ± 2.0	35.9 ± 2.0	43.3 ± 2.3	48.9 ± 2.4

Values are means ± SD. SID, strong ion difference; SID_exp_, expected plasma SID; *Z*_pH_, titrable charges.

### Experiment 3

Albumin and hemoglobin concentrations were 5.0 ± 0.2 and 13.9 ± 1.3 g/dL, respectively.

**Figure 4.**
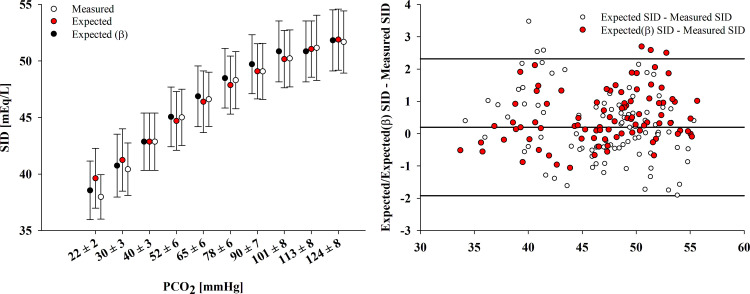
Measured vs. expected strong ion difference (SID) during respiratory acidosis (*experiment 3*). *Left*: mean values of measured and expected SID at every equilibrated CO_2_ in the control samples (i.e., with no added strong acid). The expected SID was calculated either from albumin and hemoglobin’s titrable charges (SID_exp_) or from patients’ specific whole blood buffer value β [SID_exp(β)_]. *Right*: the agreement between SID and SID_exp_, and between SID and SID_exp(β)_ is displayed through a Bland–Altman plot. The step with the PCO_2_ closest to 40 mmHg (i.e., the baseline step) was not considered in the Bland–Altman analyses, as the measured and expected SID are equal by definition.

In the control sample (Ctr), from 2 to 20% CO_2_, the measured strong ion difference (SID) increased by 12.3 ± 4.6 mEq/L. The agreement with the predicted strong ion difference was 0.2 [−1.9; 2.3] mEq/L when using SID_exp_, and 0.5 [−1.1;2.1] mEq/L when using SID_exp(β)_ (Fig. 4).

**Figure 5.**
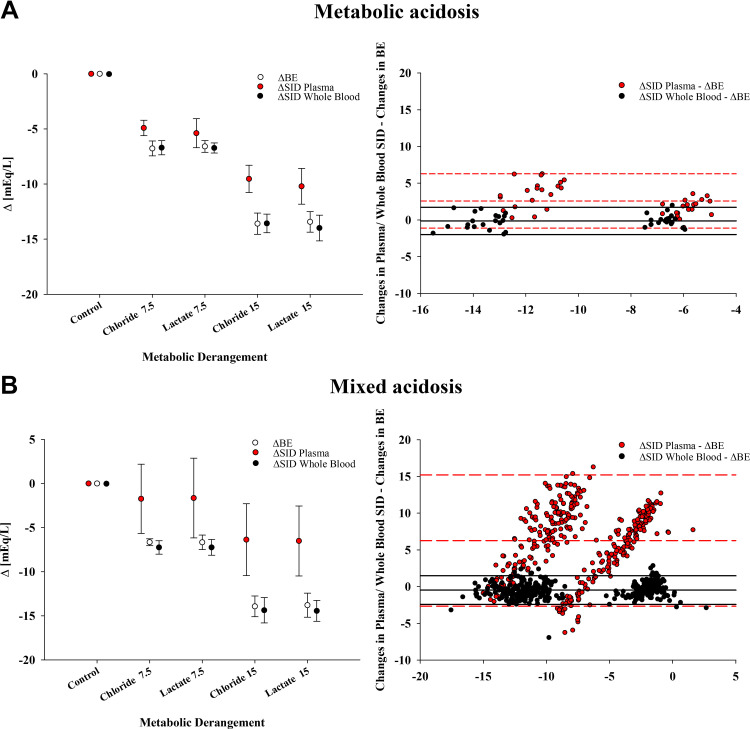
Base excess (BE), strong ion difference (SID), and whole blood SID (SID_wb_) during metabolic and mixed acidosis (*experiment 3*). This figure displays changes in base excess, plasma SID, and whole blood SID from baseline to samples with added lactate or chloride. Mean changes are displayed on the *left*, Bland–Altman analysis on the *right*. *A*: all samples are at constant equilibrated CO_2_ (metabolic acidosis), whereas in *B* samples with added lactate or chloride are at any equilibrated CO_2_ between 2 and 20% (mixed acidosis). The control sample with the PCO_2_ closest to 40 mmHg (i.e., the baseline step) was not considered in the Bland–Altman analysis, as all Δ are referred to baseline by definition.

As shown in Table 3, when compared with baseline, samples with added lactate or chloride (Lac 7.5, Cl 7.5, Lac 15, Cl 15) and constant equilibrated CO_2_ (pure metabolic acidosis) showed a decrease in BE of −6.6 ± 0.5, −6.8 ± 0.7, −13.4 ± 0.9, and −13.6 ± 1.0 mEq/L, respectively, whereas the concomitant decrease in SID was significantly lower: −5.4 ± 1.3, −4.9 ± 0.7, −10.2 ± 1.6, and −9.5 ± 1.2 mEq/L, respectively, all *P* < 0.01 (Supplemental Fig. S4; see https://doi.org/10.6084/m9.figshare.24899343.v2, for changes in single electrolytes). The agreement between ΔBE and ΔSID was weak: 2.6 [−1.1; 6.3] mEq/L. Conversely, the agreement between ΔBE and ΔSID_wb_ was strong: −0.1 [−2.0; 1.7] mEq/L (Fig. 5*A*).

**Table 3.** Metabolic acidosis at constant equilibrated CO_2_ in experiment 3

		Metabolic Acidosis (*n* = 10)			
Variable	Ctr	Lac 7.5	Cl 7.5	Lac 15	Cl 15
PCO_2_	39.9 ± 3.2	39.4 ± 3.5	40.9 ± 2.9	40.4 ± 1.8	40.5 ± 2.1
pH	7.40 ± 0.03	7.30 ± 0.02	7.29 ± 0.03	7.18 ± 0.04	7.18 ± 0.04
HCO_3_^−^	24.8 ± 1.4	19.6 ± 1.8	19.8 ± 1.7	15.3 ± 1.2	15.2 ± 1.2
Na^+^	142.9 ± 1.9	143.5 ± 1.9	144.6 ± 1.4	144.6 ± 1.4	146.2 ± 1.7
K^+^	4.0 ± 0.3	4.0 ± 0.3	4.0 ± 0.3	4.0 ± 0.3	4.2 ± 0.2
Ca^++^	1.1 ± 0.0	1.2 ± 0.0	1.2 ± 0.0	1.2 ± 0.0	1.3 ± 0.0
Mg^++^	2.0 ± 0.1	2.0 ± 0.1	2.0 ± 0.1	2.0 ± 0.1	2.0 ± 0.1
Cl^−^	106.4 ± 1.2	105.3 ± 1.1	113.2 ± 1.2	104.2 ± 0.9	119.9 ± 1.4
Lac^−^	1.6 ± 0.3	8.7 ± 0.4	1.6 ± 0.4	15.8 ± 0.7	1.5 ± 0.3
SID	42.9 ± 2.5	37.5 ± 2.5	38.0 ± 2.4	32.7 ± 2.0	33.3 ± 2.7
β	28.5 ± 2.8	30.6 ± 2.5	31.1 ± 2.6	33.1 ± 2.9	33.7 ± 2.8
BE	0.0 ± 1.5	−6.6 ± 1.7	−6.8 ± 1.8	−13.4 ± 2.2	−13.6 ± 2.1
ΔSID	/	−5.4 ± 1.3	−4.9 ± 0.7	−10.2 ± 1.6	−9.5 ± 1.2
ΔBE	/	−6.6 ± 0.5	−6.8 ± 0.7	−13.4 ± 0.9	−13.6 ± 1.0
ΔSID_wb_	/	−6.7 ± 0.5	−6.7 ± 0.7	−14.0 ± 1.2	−13.5 ± 0.8

Values are means ± SD. BE, base excess; SID, strong ion difference; SID_wb_, whole blood SID; /, not applicable.

When comparing baseline with any other sample (Lac 7.5, Lac 15, Cl 7.5, Cl 15) at any equilibrated CO_2_ from 2 to 20% (mixed acidosis), the overall agreement between ΔBE and ΔSID was very weak (6.3 [−2.7; 15.2] mEq/L), whereas between ΔBE and ΔSID_wb_ it remained strong: −0.5 [−2.4; 1.5] mEq/L (Fig. 5*B*).

### Simplified Model

Results are presented in Supplemental Fig. S5 (see https://doi.org/10.6084/m9.figshare.24899337.v2): Supplemental Fig. S5, *A* and *B* refers to all experiments during respiratory acidosis. The agreement between SID and SID_exp(CLSI)_ was strong: 0.5 [−2.3; 3.3] mEq/L, with only 16/258 comparisons (6.2%) showing a bias ≥ 3 mEq/L (Supplemental Fig. S5*A*). The bias was proportional to albumin’s concentration (*P* < 0.01, Pearson’s *r* 0.35, Supplemental Fig. S5*B*). Supplemental Fig. S5, *C* and *D* refers to *experiment 3*: during pure metabolic acidosis (Supplemental Fig. S5*C*), the agreement between ΔBE_CLSI_ and ΔSID was weak (1.9 [−1.1; 5.0] mEq/L), whereas between ΔBE_CLSI_ and ΔSID_wb(CLSI)_ it was strong: −0.1 [−1.9;1.7] mEq/L. During mixed acidosis (Supplemental Fig. S5*D*), the agreement between ΔBE_CLSI_ and ΔSID worsened (5.1 [−2.5; 12.7] mEq/L), whereas between ΔBE_CLSI_ and ΔSID_wb(CLSI)_ it remained strong: −0.5 [−2.4; 1.3] mEq/L.

## DISCUSSION

The results of the present study can be divided into:

1)  *Physiological Findings*: changes in plasma SID due to redistribution of strong ions at varying pH can be predicted from albumin and hemoglobin’s titrable charges, i.e., from the noncarbonic buffer value of whole blood.
2)  *Clinical Implication*: only changes in plasma SID that are not explained by redistribution of strong ions are paralleled by equal changes in BE.

### Physiological Findings

#### Isolated plasma.

In 1992, Fogh-Andersen et al. (14) demonstrated that the total net charge on albumin was more negative than the charge of its lateral aminoacidic residues, due to an excess of bound chloride with respect to positive electrolytes. He also found that, at varying pH, changes in albumin’s bound-electrolytes tended to preserve the total net charge on the protein by compensating for the change in its titrable charge *Z*_pH_ (14) (Supplemental Fig. S1, see https://doi.org/10.6084/m9.figshare.24903384). Based on Fogh-Andersen’s data, we hypothesized that pH-induced changes in SID in isolated plasma would be predictable from changes in albumin’s *Z*_pH_. The results of *experiment 1* corroborated this hypothesis in both healthy subjects and patients .

Of note, Fogh-Andersen’s data predicted only changes in chloride and calcium at varying pH (Fig. 1). Here, we also found changes in sodium (Supplemental Fig. S3*A*, see https://doi.org/10.6084/m9.figshare.24899334.v2), as previously reported by others in similar experiments: Staempfli and Constable (35) ascribed them to measurements artifacts; others suggested instead that sodium-albumin binding and/or water-albumin binding at varying pH would be more reasonable explanations (36).

#### Whole blood.

The distribution of electrolytes between plasma and red blood-cells is a function of the electrical charge of compartmentalized molecules (essentially albumin and hemoglobin), as determined by the Donnan theory (37). Accordingly, in an isolated red blood-cells experiment, Dalmark (17) found that changes in the intracellular concentration of chloride at varying pH were solely determined by hemoglobin’s buffer value, i.e., by the change in its titrable charge. Combining the models of Fogh-Andersen et al. (14) and Dalmark (17), we have demonstrated that the overall change in plasma SID during whole blood CO_2_ tonometry can be predicted from albumin and hemoglobin’s titrable charges: in all our experiments, the agreement between the expected and measured SID was strong across a wide pH range. *Experiments 1* and *2* also showed that hypoalbuminemia and/or anemia are associated with lower changes in plasma SID at varying pH, further corroborating our hypothesis.

Confirmation of the major role of albumin and hemoglobin in driving electrolytes redistribution at varying pH can also be found in the study by Reeves (38), pioneer of the alpha-stat theory. This author demonstrated that when blood’s pH is altered by temperature variations no redistribution of electrolytes occurs (39), as imidazoles ionization remains unchanged (i.e., *Z*_pH_ is constant).

An intuitive explanation to the observed relationship between albumin and hemoglobin’s titrable charges and electrolytes’ redistribution can be given based on bicarbonate kinetics and the law of electroneutrality: briefly, albumin and hemoglobin are responsible either for the increase in plasma bicarbonate during respiratory acidosis, or for blunting its reduction during metabolic acidosis (40). As detailed later, under both circumstances, the exceeding negative charge of bicarbonate must be balanced by an equal increase in plasma positive charges. This can only be obtained by a displacement of strong ions, increasing the SID.

##### Respiratory acidosis.

Carbon dioxide equally diffuses in plasma and red cells, and it is mainly buffered by albumin and hemoglobin leading to an increase in bicarbonate (41). In isolated plasma, the negative charge of bicarbonate is initially balanced by an equal increase in the positive titrable charge (*Z*_pH_) on albumin’s imidazoles (3), which in turn drives an equal binding of electrolytes to preserve the total net charge on the protein as per Fogh-Andersen’s model (14) (Supplemental Fig. S1, see https://doi.org/10.6084/m9.figshare.24903384). This process is paralleled, in whole blood, by an increase in erythrocytes’ bicarbonate determined by hemoglobin’s buffer value (26). The increasing red cells’ bicarbonate then shifts to plasma where, for electroneutrality to be preserved, an equal increase is SID must occur. The latter is obtained through a shift of chloride and water into the erythrocytes, decreasing the plasma concentration of chloride and increasing that of cations (16) (Supplemental Fig. S3*B*, see https://doi.org/10.6084/m9.figshare.24899334.v2). The overall effect of respiratory acidosis in whole blood is therefore that Δ_(plasma)_$\mathrm{HC}O_{3}^{-}$ = Δ_(Albumin)_*Z*_pH_ + Δ_(Hemoglobin)_*Z*_pH_ = Δ_(plasma)_SID. This one-to-one increase in bicarbonate and SID at varying CO_2_ was recently confirmed in vivo by our group (20).

##### Metabolic acidosis.

Addition of lactate or chloride to blood is initially buffered in plasma by bicarbonate and, to a lesser extent, by albumin (40). As aforementioned, the increasing positive albumin’s titrable charge (*Z*_pH_) drives an equal binding of electrolytes following Fogh-Andersen’s model (14). The CO_2_ formed in plasma by the bicarbonate buffer system then shifts into red cells, where it regenerates bicarbonate in an amount that depends on hemoglobin’s buffer value (42, 43). The newly formed red cells’ bicarbonate moves back to plasma where its increasing negative charge must be balanced by an equal increase in SID. This is obtained through a shift of chloride, water and of the added ion itself, if permeant, into the erythrocytes (Supplemental Fig. S4, see https://doi.org/10.6084/m9.figshare.24899343.v2). The overall effect of metabolic acidosis in whole blood is therefore that Δ_(plasma)_Acid^−^ − Δ_(plasma)_HCO_3_^−^ = Δ_(Albumin)_*Z*_pH_ + Δ_(Hemoglobin)_*Z*_pH_ = Δ_(plasma)_SID.

#### Noncarbonic whole blood buffer value (β).

The titrable charge on albumin and hemoglobin (*Z*_pH_) is not routinely calculated in clinical practice. However, these proteins are the main determinants of the noncarbonic buffer value of whole blood (β) (26), and, in *experiment 3*, we have shown that patient’s specific β can be used to predict pH-induced changes in plasma SID with equivalent accuracy compared with *Z*_pH_. Interestingly, the prediction remained clinically acceptable when using β_CLSI_ in our simplified model. This broadens the clinical applicability of our findings, as CLSI standards are currently used by bedside blood gas analyzers. As expected, the accuracy of the simplified model worsened with hypoalbuminemia (Supplemental Fig. S5*B*, see https://doi.org/10.6084/m9.figshare.24899337.v2), as CLSI assumes normal values of plasma proteins. However, the bias remained largely acceptable from a clinical standpoint.

It can be concluded that pH-induced changes in plasma SID are a function of the noncarbonic whole blood’s buffer value, i.e., of the change in albumin and hemoglobin’s titrable charge at varying pH.

### Clinical Implications

The possibility of predicting changes in SID at varying pH finds a strong clinical implication in the interpretation of BE. Indeed, BE reflects changes in whole blood SID (24), which cannot be directly measured, but that our model allows to estimate indirectly with two simple steps: first, the difference between the measured and expected plasma SID reflects the amount of acid added to plasma; second, conversion to whole blood is possible by means of the distribution ratio of bicarbonate (Eq. *7*). The assumption is that lactate and chloride, the most common measurable metabolic acids in clinical practice, distribute between plasma and red cells in the same manner as bicarbonate, as previously shown (44, 45). The validity of this model is demonstrated by *experiment 3*, showing a very strong agreement between changes in BE and in our calculated whole blood SID during both metabolic and mixed acidosis.

**Figure 6.**
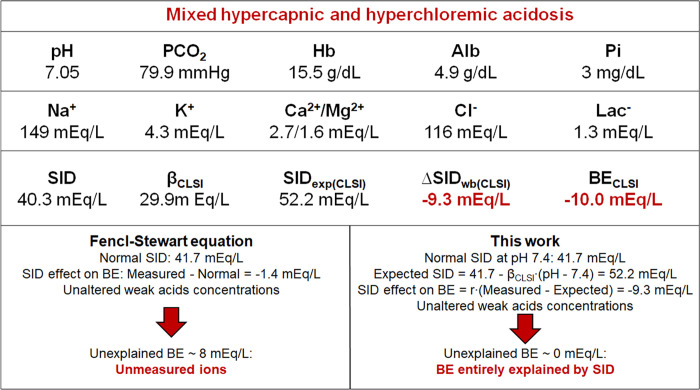
Fencl–Stewart equation vs. our simplified model applied to mixed acidosis. Data refer to whole blood of one of the healthy subjects from *experiment 3* after addition of hydrochloric acid (∼15 mEq/L) and equilibration with ∼15% CO_2_. As shown, ignoring electrolytes redistribution, the Fencl-Stewart equation underestimates the effect of strong ions on base excess (BE), erroneously implying the presence of unmeasured ions.

Conversely, changes in BE and plasma SID agreed very poorly, especially during mixed disturbances, where differences up to 15 mEq/L were observed. This corroborates the hypothesis that variations in the plasma concentrations of strong ions do not reflect changes in the net pool of whole blood charges, and, thereby, should not be considered as proxy of BE. Our findings are at variance with current models attempting to partition BE into plasma components using the Fencl-Stewart approach (4–9): these models consider that any deviation in SID from a fixed, reference value [e.g., 41.7 mEq/L (3)] is paralleled by an equal BE. On the contrary, we have demonstrated that part of the change in SID during acidosis reflects redistribution of electrolytes that does not alter the whole blood SID, and thereby the BE. A practical example is given in Fig. 6, where we applied our model and the Fencl–Stewart equation to whole blood of one of the healthy subjects from *experiment 3*, with in vitro-induced mixed hypercapnic and hyperchloremic acidosis (pH 7.05, PCO_2_ 79.9 mmHg, [Cl^−^] 118 mEq/L). As shown, despite the hyperchloremia with reduced BE (−10 mEq/L), the plasma SID is almost normal (40.3 mEq/L). Since the concentration of total weak acids (albumin and phosphate) is also normal, the Fencl–Stewart equation concludes that unmeasured ions (UI) are present to explain the BE. Conversely, our simplified model calculates a change in whole blood SID of −9.3 mEq/L, confirming that the BE is entirely explained by SID variations. This is likely the reason why the Fencl–Stewart equation might be inaccurate in estimating UI when compared with the reference method, i.e., the strong ion gap (34). It must be acknowledged, however, that the Fencl–Stewart approach is intended for a bedside evaluation of acid-base disturbances, and, despite its simplifying assumptions, it carries undoubtful clinical benefits in this regard (5). Moreover, as expanded in *Limitations*, it uses the base excess of the extracellular fluid (4) rather than whole blood base excess, and application of our in vitro findings to the extracellular space requires validation.

### Limitations

Some limitations must be highlighted: first, our model relies on the calculation of SID, which might be affected by measurements’ inaccuracies depending on the type of blood gas analyzer used (46). The fact that two different machines were used in this study (one for *experiments 1* and *2*, and a different one for *experiment 3*) with very similar results alleviates our concerns in this regard; second, magnesium concentration was only assessed at baseline and considered constant throughout the experiments: while we acknowledge that binding to albumin (15) and transerythrocyte movements of water might affect magnesium concentration at varying pH, we do not believe that this altered our results in a quantitatively significant manner; third, our model does not take into consideration phosphate species: both inorganic phosphate in plasma and 2,3 bisphosphoglycerate in red cells contribute to whole blood buffering (26), and the former also freely moves between plasma and red-cells (47). However, the quantitative role of these species is small (26), and their concentration is hardly ever available in clinical practice. Accordingly, their inclusion in this model would reduce its clinical applicability while only minimally improving its accuracy. This is demonstrated by the results relative to SID_exp(β)_ in Fig. 4, where accounting for the contribution of phosphate species through the whole blood buffer value β did not significantly change our results; finally, our results are limited by the in vitro nature of the experiments, and in vivo applicability remains to be demonstrated. Importantly, the in vivo PCO_2_-independent parameter of acute metabolic acidosis is the extracellular, or standard, base excess (SBE) rather than whole blood BE (20, 48). As the extracellular buffer value β is lower than in whole blood (26), changes in plasma SID at varying pH are expected to be lower in vivo than in vitro. Accordingly, we have recently shown that pH-dependent changes in plasma SID during in vivo acute respiratory acidosis equal changes in plasma bicarbonate (20), which are a function of the extracellular buffer value β. A significant redistribution of strong ions has also been previously reported during in vivo acute metabolic acidosis (21, 23). It is important to point out that this study does not suggest using whole blood BE rather than SBE in clinical practice. However, the in vitro nature of the experiments did not allow us to calculate SBE, nor to validate our prediction of changes in plasma SID for in vivo acidosis. This is currently being investigated by our group as a future project.

### Conclusions

During experimental acidosis, changes in plasma SID reflecting electrolytes redistribution can be predicted from concomitant changes in albumin and hemoglobin’s titrable charges or, simpler, from the noncarbonic buffer value of whole blood (β). Expected changes in SID can be used to assess the actual contribution of strong ions to base excess, allowing an accurate interpretation of this widely used indicator of metabolic acidosis. All variables used in this model are available in clinical practice, including β_CLSI_, which is currently incorporated in blood gas analyzers for the calculation of BE (32). This is a great advantage with respect to previous comprehensive acid base models, which require complex calculations limiting clinical applicability (11, 24). Validation of our findings in vivo might improve the bedside understanding of metabolic acidosis.

## DATA AVAILABILITY

The complete data set is available at https://doi.org/10.6084/m9.figshare.24903423.

## SUPPLEMENTAL DATA

10.6084/m9.figshare.24903429.v1 Supplemental Material: https://doi.org/10.6084/m9.figshare.24903429.v1.

10.6084/m9.figshare.24903384 Supplemental Fig. S1: https://doi.org/10.6084/m9.figshare.24903384.

10.6084/m9.figshare.24903414 Supplemental Fig. S2: https://doi.org/10.6084/m9.figshare.24903414.

10.6084/m9.figshare.24899334.v2 Supplemental Fig. S3: https://doi.org/10.6084/m9.figshare.24899334.v2.

10.6084/m9.figshare.24899343.v2 Supplemental Fig. S4: https://doi.org/10.6084/m9.figshare.24899343.v2.

10.6084/m9.figshare.24899337.v2 Supplemental Fig. S5: https://doi.org/10.6084/m9.figshare.24899337.v2.

## DISCLOSURES

No conflicts of interest, financial or otherwise, are declared by the authors.

## AUTHOR CONTRIBUTIONS

L.G., P.C., E.P., L.G., and T.L. conceived and designed research; F.Z., S.B., M.K., and P.B. performed experiments; L.G. analyzed data; L.G., F.Z., M.B., F.D., A.Z., P.C., E.P., and T.L. interpreted results of experiments; L.G., F.Z., G.D.S., and A.D.M., prepared figures; L.G. drafted manuscript; L.G., F.Z., M.B., G.D.S., S.B., M.K., F.D., P.B., A.Z., A.D.M., P.C., E.P., L.G., and T.L. edited and revised manuscript; L.G., F.Z., M.B., G.D.S., S.B., M.K., F.D., P.B., A.Z., A.D.M., P.C., E.P., L.G., and T.L approved final version of manuscript.
